# Oxymatrine suppresses the growth and invasion of MG63 cells by up-regulating PTEN and promoting its nuclear translocation

**DOI:** 10.18632/oncotarget.17783

**Published:** 2017-05-10

**Authors:** Ming He, Linlin Jiang, Bin Li, Guangbin Wang, Jiashi Wang, Yonghui Fu

**Affiliations:** ^1^ Department of Orthopedic Surgery, Shengjing Hospital of China Medical University, Shenyang, Liaoning, People’s Republic of China; ^2^ Department of Electrotheropy, Shenyang Medical College Affiliated Central Hospital, Shenyang, Liaoning, People’s Republic of China

**Keywords:** oxymatrine, MG63 cells, PTEN, signaling pathway, PI3K/Akt

## Abstract

Studies demonstrated that reduced PTEN levels are associated with poor prognoses of osteosarcoma. The nuclear localization of PTEN is important for its tumor suppressive function. Equally importantly, PTEN is the most significant negative regulator of PI3K/Akt signaling cascade, the constitutively activated pathway in osteosarcoma. In our study MG63 cells and U2OS cells were treated with the indicated concentrations of oxymatrine, in order to find the inhibition of oxymatrine to cells. We found the functions of oxymatrine on proliferation, apoptosis and invasion in cells. Oxymatrine could increase the expression of PTEN and promote its nuclear translocation in MG63 cells. In addition, oxymatrine could induce cell cycle arrest in G1 phase and apoptosis of MG63 cells. The migration and invasion potential of MG63 cells were also markedly inhibited by oxymatrine. Oxymatrine could suppress the growth and invasion of MG63 human osteosarcoma cells by up-regulating PTEN and promoting its nuclear translocation and inhibiting PI3K/Akt signaling pathway.

## INTRODUCTION

Osteosarcoma (OS) is the most primary bone tumor and occurs predominantly in adolescents and young adults [1, 2]. Traditional therapeutic for osteosarcoma approaches include local control of the primary lesion by surgery or chemotherapy, and treatment of disseminated disease with multiagent cytotoxic chemotherapy [3].People tried their best to advance osteosarcoma therapy over the past several decades to enhance patient outcomes, however, over the last decades, there have been no noticeable improvements in patient survival [1, 3, 4].Therefore, the identification of the effector molecules and signal pathways responsible for regulating chemotherapy resistant and malignant development is crucial for improving the osteosarcoma treatment level [3]. The genetic alterations of osteosarcoma include the inactivation of tumor suppressor genes and activation of oncogenes. e.g., mutations of P53, PTEN, EGFR, Ras and their aberrant effect on signaling pathway have been thoroughly characterized [5].

As a tumor suppressor gene, *pten* was identified in 1997 which is altered in various types of solid tumors, mainly including osteosarcoma, melanoma, breast, prostate, endometrial cancer [6]. PTEN is one of the most commonly tumor suppressor in human cancers, which is a central negative regulator of thePI3K (phos-phoinositide-3 kinase)/Akt signaling pathways for cell growth, metabolism, proliferation and survival [7, 8]. PTEN has very distinct roles in the cytoplasm and the nucleus. Generally, in the primary, differentiated, and resting cells, PTEN is predominantly localized cell nucleus, while cytoplasmic PTEN is predominately found in neoplastic tissues. In the nucleus, PTEN displays a PI3K-independent manner and plays tumor suppressor role [8, 9]. The absence of nuclear PTEN is associated with more aggressive carcinoma and serves as a prognostic indicator [10].

**Figure 1:**
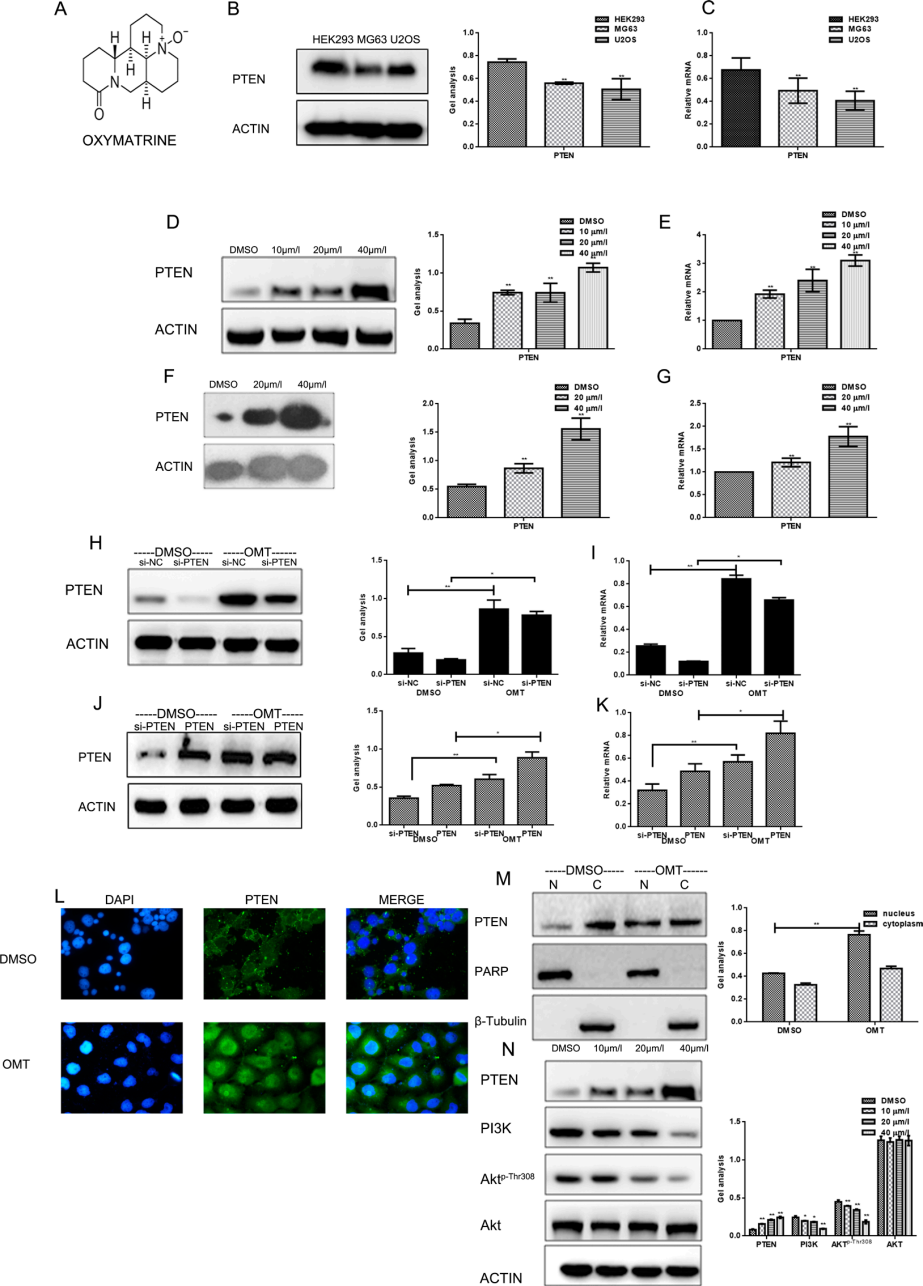
OMT increased the expression of PTEN (**A**) The chemical structure of OMT. (**B**, **C**) The expression of PTEN was detected in HEK293, MG63 and U2OS cells. (**D**, **E**) MG63 cells were treated with DMSO alone or indicated concentration of OMT for 24 h, the indicated proteins and mRNA level were detected by western blot analysis and real-time RT-PCR respectively, Data are shown as mean ± SEM. (**F**, **G**) U2OS cells were treated with DMSO alone or indicated concentration of OMT for 24 h, the indicated proteins were detected by western blot analysis. (**H**, **I**) MG63 cells transfected with si-PTEN or si-scramble (negative control) and then treated with or without OMT, the indicated proteins and mRNA level were detected by western blot analysis and real-time RT-PCR respectively, Data are shown as mean ± SEM. (**J**, **K**) MG63 cells transfected with Flag-PTEN after silenced PTEN, then treated with or without OMT. The indicated proteins and mRNA level were detected by western blot analysis and real-time RT-PCR respectively. Data are shown as mean ± SEM. (**L**) Immunofluorescence staining of PTEN (green) in MG63 cells. DAPI (blue) is for nucleus Original magnification, × 400. (**M**). Detection of PTEN in nucleus and in cytoplasm respectively. PARP as the nuclear marker, and β-tubulin as the cytoplasmic marker. (**N**) The indicated proteins were detected by western blot in MG63 cells.

Oxymatrine [11] (chemical structure shown in Figure 1A), is a primary component of the dried root of Sophora flavescensAiton, which is a herb medicine could be found widely in China, Japan and some European countries [12]. It has been studied in a variety of tumor cells lines and xenografts mice, such as breast, lung, gastric, melanoma, leukemia, cervix, pancreatic and hepatocellular carcinoma [13]. But the study of OMT on human osteosarcomas has not been reported. It has been reported that OMT exerted antitumor effect on different tumor cells through various mechanisms. For instance, OMT potently inhibited SGC996 gallbladder tumor cells growth in nude mice by up-regulating the activated Caspase3 and Bax and down-regulating Bcl-2 and NF-κB. In addition, OMT inhibited the proliferation and induced apoptosis of human hepatoma SMMC-7721 cells by cell cycle blockage in G2/M and S phase [14]. Another study indicated that, OMT induced apoptosis by activating the Caspase9/Caspase3-mediated intrinsic pathway in HL-60 cells and A375 cells [15, 16]. Zou et al. reported that OMT killed colon cancer SW1116 cells by inhibiting telomerase activity [17]. Additionally, it was reported that the inhibitory effects of OMT on MCF-7 cells may be due to the inhibition of SP and Wnt/β-catenin signaling pathway [18].

In this study, we found that OMT suppresses the proliferation and invasion of MG63 cells, and promotes its apoptosis through up-regulating the expression of PTEN, promoting its nuclear translocation and inhibitingPI3K/Akt pathway.

## RESULTS

### OMT inhibits PI3K/Akt cascade by up-regulating the expression of PTEN and promoting its nuclear translocation and pathway

Firstly, we detected the content of PTEN in different cell lines (Figure 1B, 1C). The content of PTEN in tumor cells was lower than that in HEK293 cells. We detected the effects of OMT on the expression of PTEN. By real time RT-PCR and western blot analysis, we found that treatment of OMT could markedly increase the expression of PTEN in concentration dependent manner in MG63and U2OS cells (Figure 1D–1G). To further verify the specific targeting of OMT on PTEN, we performed two group experiments. Firstly, MG63 cells were transfected with si-PTEN and si-scramble (negative control) respectively, and treated with or without OMT. The expressions of PTEN were tested by western blot analysis and real time RT-PCR (Figure 1H, 1I). Data showed that OMT could significantly promote the expression of PTEN in both protein and mRNA levels in the OMT treated groups compared with the DMSO control groups. Besides, in MG63 cells, we over-expressed PTEN after silence PTEN. Then cells were treated with or without 20 μM OMT for 24h.The results showed that the increased degree of PTEN in cells treated with OMT was similar with that of cells silenced PTEN in both protein and mRNA levels (Figure1J, 1K), which indicated that OMT could promote endogenous PTEN expression.

Next, we studied the effect of OMT on the distribution of PTEN in cells. As showed in Figure 1L, PTEN mainly localized in cytoplasm when treated with DMSO, while after 24 h treated by OMT, PTEN obviously expressed in the nucleus, meanwhile, the expression of PTEN was significantly increased. Subsequently, we separated the nucleus and the cytoplasm to detect the distrubution of PTEN. Results showed that the expression of PTEN in the nucleus was significantly increased after treated by OMT (Figure 1M).

Since PTEN is the major negative regulator of PI3K/Akt signaling pathway and OMT could increase the expression of PTEN, so, whether OMT could play a role in PTEN/PI3K/Akt. So PI3K, Akt and the phosphorylation degree of Akt (Thr308) were detected by western blot analysis. After exposed to the indicated concentrations of OMT for 24 h, as shown in Figure 1N, following the increased concentration of OMT, PTEN expression increased, both PI3K and Akt-p-Thr308decreased, but the total level of Akt did not change, which indicated that OMT could inhibit PI3K/Akt pathway by increased PTEN.

### OMT inhibits the growth of human osteosarcoma cells by cell cycle arrest

**Figure 2:**
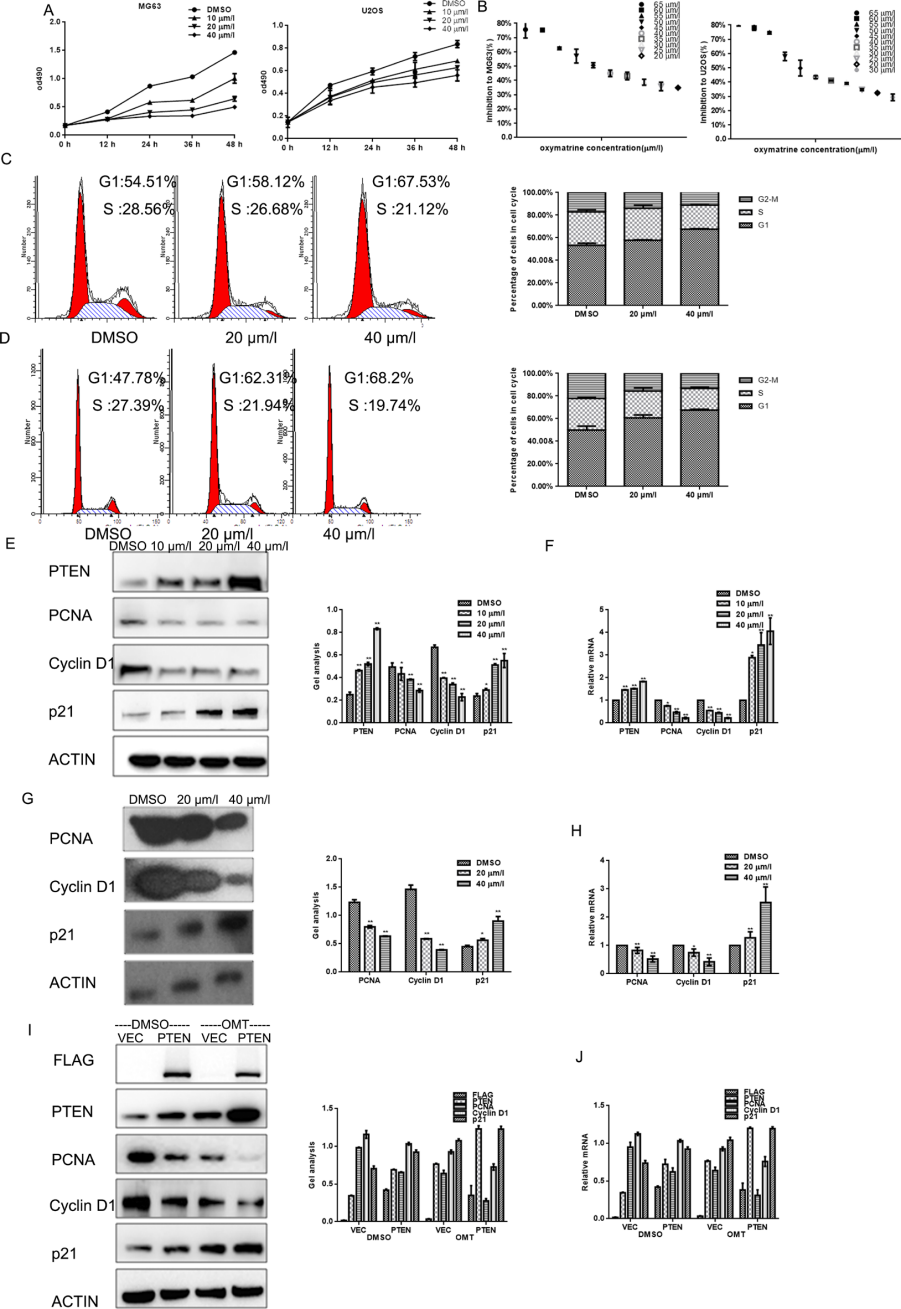
OMT inhibits the growth of MG63 cells (**A**) MG63and U2OS cells were treated with indicated concentrations of OMT, cells growth was detected by MTT assay. Data are shown as mean ± SEM. (**B**) IC50 of MG63 and U2OS cells was detected by MTT assay. Data are shown as mean ± SEM. (**C**, **D**) MG63and U2OS cells were analyzed by a FACS vantage flow cytometer with the Cell Quest acquisition and analysis software program (Becton Dickinson and Co., San Jose, CA), the experiment was repeated three times. (**E**, **F**) MG63 cells were treated with the indicated concentration of OMT for 24 h, the indicated proteins and mRNA levels were detected by western blot and real-time RT-PCR. Data are shown as mean ± SEM.*P < 0.01 vs. DMSO treated group. (**G**, **H**) U2OS cells were treated with the indicated concentration of OMT for 24 h, the indicated proteins were detected by western blot.(**I**, **J**) MG63 cells transfected with Flag-PTEN or Flag-vector then treated with or without OMT. The indicated proteins were detected by western blot analysis and real-time RT-PCR. Data are shown as mean ± SEM.*P < 0.01 vs. PTEN/vector.

Next, the inhibitory effects of OMT on growth of MG63and U2OScells were detected. By MTT assay, the proliferations of cells were suppressed in a concentration dependent manner (Figure 2A).Data showed that the 24 h IC_50_ value of OMT for MG63 cells and U2OS cells were 47.75 μM and 44.58 μM respectively (Figure 2B).In order to clarify the inhibitory mechanism of OMT on cell growth, cell cycle analysis was performed afterMG63and U2OScells were treated with the indicated concentrations of OMT for 24 h. Data showed that these two cell lines were both arrested in G1 phase and the cell percentage in S phase decreased (Figure 2C, 2D).

Since p21, Cyclin D1 and PCNA are the key regulators in G_1_ phase of the cell cycle; we examined the expression of them by real time RT-PCR and Western blot analysis. Results showed that the degree of PCNA and Cyclin D1 decreased and p21 was increased significantly after exposed to OMT for 24 h at the indicated concentration in MG63 and U2OS cells (Figure 2E–2H).To further confirm the involvement of OMT in cell cycle arrest is through PTEN, MG63 cells were transfected with PTEN or vector as control, the three regulators were detected by western blot. We found that PCNA and Cyclin D1 decreased and p21 increased in both the PTEN over-expressed group and the OMT treated groups (Figure 2I, 2J).

### OMT induces apoptosis of osteosarcoma cells

**Figure 3:**
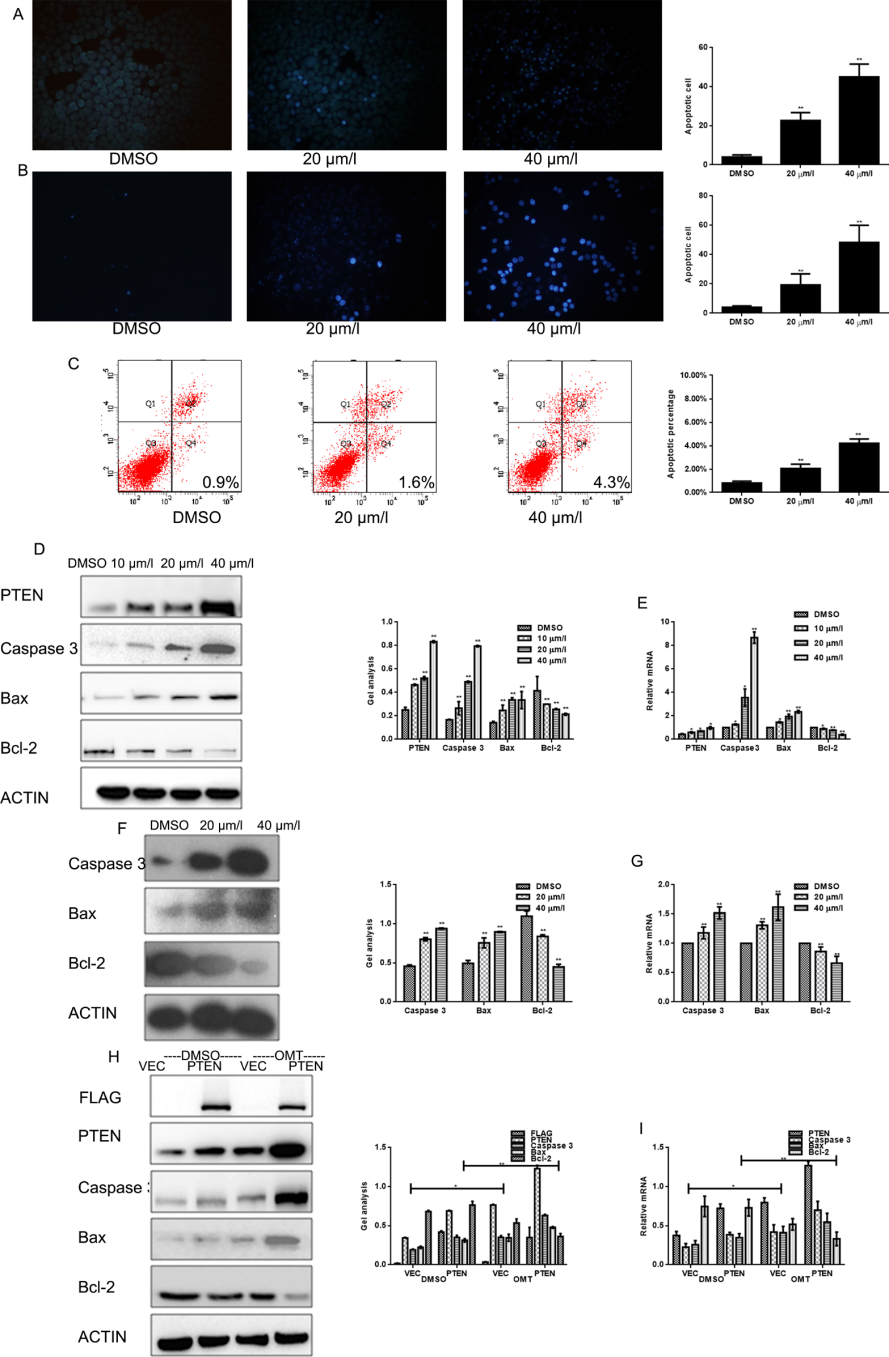
OMT promotes the apoptosis of MG63 (**A**, **B**) MG63 and U2OScells were treated with OMT for 24 h and stained with Hochest33258. (**C**) MG63 cells were treated with OMT for 24 h then cells were testedby ANNEXIN-V-FITC apoptosis detection kit and analyzed by FCAS. The experiment was repeated for three independent times. (**D**, **E**) MG63 cells were treated with DMSO alone or indicated concentration of OMT for 24 h, the indicated proteins and mRNA level were detected by western blot analysis and real-time RT-PCR respectively, Data are shown as mean ± SEM. *P < 0.01 vs. DMSO treated group; (**F**, **G**) U2OS cells were treated with DMSO alone or indicated concentration of OMT for 24 h, the indicated proteins were detected by western blot analysis. (**H**, **I**) MG63 cells transfected with Flag-PTEN or Flag-vector, and then treated with or without OMT. The indicated proteins were detected by western blot analysis Data are shown as mean ± SEM.

To test whether OMT could induce apoptosis of MG63 cells, Hochest 33258 staining assay and Annexin-V-FITC apoptosis detection were performed. As shown in Figure 3, condensed chromatin was observed in OMT treated MG63 and U2OS cells, and apoptosis rate was increased in a OMT dependent-manner (Figure 3A–3C). Next, the apoptosis-associated proteins were detected by western blot and real time RT-PCR. Date showed that expression of caspase-3 and Bax increased and Bcl-2 decreased in OMT concentration-dependent manner in MG63 and U2OS cells (Figure 3D–3G).Similarly, these indexes were detected in the PTEN over-expressed groups. As shown in Figure 3H and 3I, results showed that both over-expressed PTEN and treated by OMT could increase the expression of caspase-3 and Bax, and decrease Bcl-2 level. These data indicated that OMT induced apoptosis by regulating apoptosis-associated regulators.

### OMT represses the migratory and invasive potential of osteosarcoma cells

**Figure 4:**
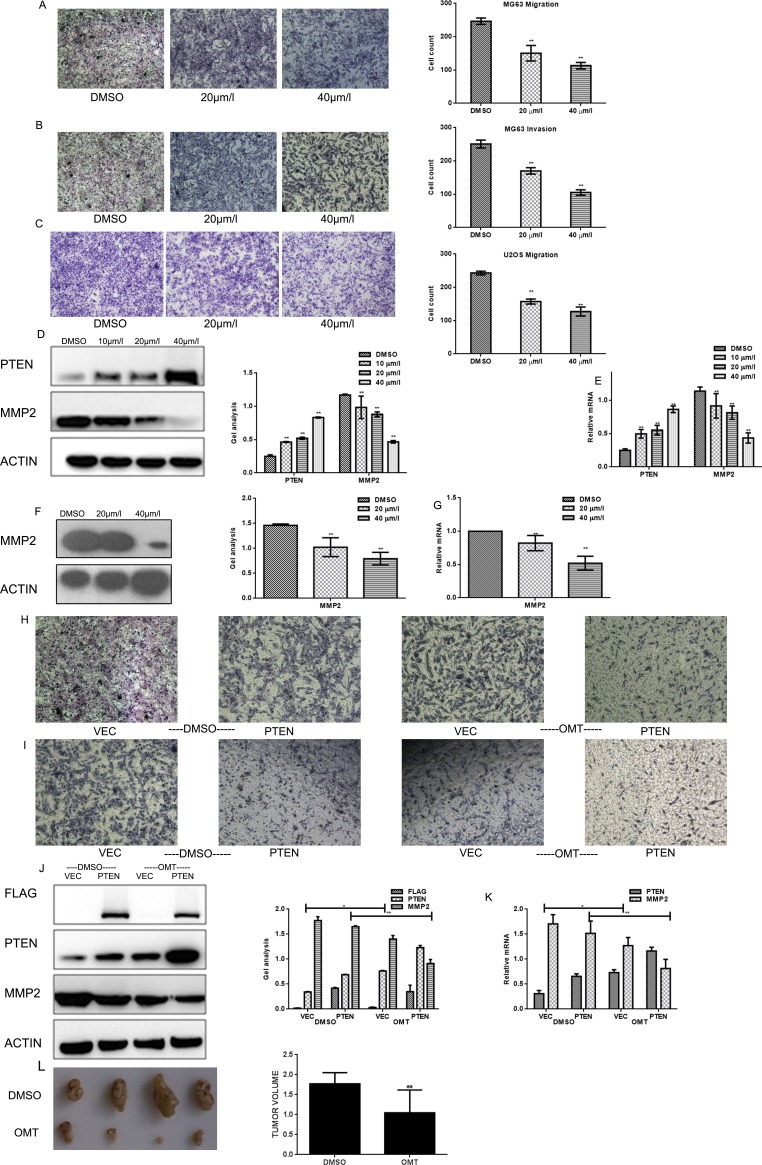
OMT represses the migratory and invasive potential of MG63 (**A**, **B**) MG63 cells were treated with OMT for 24 h; transwell assay with or without matrigel was performed. Cells were counted and results represent the mean ± SD of three experiments. *P < 0.01 vs. DMSO treated group. (**C**) U2OS cells were treated with OMT for 24 h; transwell assay without matrigel was performed. Cells were counted and results represent the mean±SD of three experiments. *P < 0.01 vs. DMSO treated group. (**D**, **E**) MG63 cells were treated with DMSO alone or indicated concentration of OMT for 24 h, the indicated proteins and mRNA level were detected by western blot analysis and real-time RT-PCR respectively, Data are shown as mean ± SEM. *P < 0.01 vs. DMSO treated group; (**F**, **G**) U2OS cells were treated with DMSO alone or indicated concentration of OMT for 24 h, the indicated proteins were detected by western blot analysis and real-time RT-PCR. (**H**, **I**) The metastasis and invasion potential of indicated group were detected by transwell assay. The experiment was repeated for three independent times. (**J**, **K**) MG63 cells transfected with Flag-PTEN or Flag-vector then treated with or without OMT. The indicated proteins were detected by western blot analysis. Data are shown as mean ± SEM. (**L**) Photographic illustration of tumors from control and oxymatrine-treated nude mice on the day of sacrifice (day 16) and the tumor volume of them were showed in the graph.

To study whether OMT is involved in suppressing migration and invasion of human osteosarcoma cells, transwell assay (with or without matrigel) were performed. Results showed that OMT significantly decreased the invasion and migration potential of MG63 cells in a dose-dependent manner (Figure 4A–4C). Subsequently, MMP2, the indicator of invasion and metastasis was tested at protein and mRNA levels respectively. With the increasing concentration of OMT, MMP2 expression significantly decreased, which implied OMT inhibited the invasion and metastasis potential of MG63and U2OS cells (Figure 4D–4G). Additionally, transwell assay (with or without matrigel) were also performed in MG63cells transfected with or without Flag-PTEN, then cells were treated with or without OMT. As shown in Figure 4H, 4I, the metastasis and invasion potential of the cells were significantly attenuated in both over-expressed Flag-PTEN but without OMT treatment cells and the cells expressed Flag-vector and treated with OMT. Similarly, the expression of MMP2 decreased in these two groups cells compared with cells expressed Flag-vector and without OMT treated (Figure 4J, 4K).These data demonstrated OMT could decrease MMP2 expression and suppress migration and invasion of MG63cells and U2OS cells.

### OMT inhibits tumor growth *in vivo*

In order to detect the inhibitory effect of OMT on MG63 cells *in vivo*, we performed elementary tumor xenografts in nude mice. In these 16 days, each group sacrificed one mouse. On the day of sixteenth, the weights of the tumors were weighed after dissection. As shown in Figure 4L, 100 mg/kg OMT treatments resulted in about 44.01% tumor suppression compared with the saline group.

## DISCUSSION

As a classical tumor suppressor, PTEN is the most significant negative regulator of the PI3K signaling pathway. PTEN loss contributes to activate the PI3K/AKT signaling pathway and consequent tumor genesis. Studies have shown that there were abnormal expression levels of the phosphate and tension homolog (PTEN) gene in human osteosarcoma cells or tissues [19].

The nuclear import of PTEN is important in cell cycle regulation [10, 20].Studies reported that nuclear PTEN could facilitate G1 arrest and increase apoptosis in HEK293 cells [21, 22]. Radu et al. reported that PTEN decreased the levels and nuclear localization of cyclin D1 and induced the growth arrest [23]. These data suggests that the regulation of PTEN shuttle between the nucleus and cytoplasm contributes to its tumor suppressor function. MG63 cells transfected with PTEN gene were susceptible to apoptosis. Apoptosis induced by PTEN is dependent on PI3K/Akt signaling [24].

Akt is also an important target of PTEN, import of PTEN into PTEN-deficient tumor cells such as glioma, breast, and prostate cancer cells resulted in a decrease of activated Akt. Phosphorylation of Akt-Thr308 is due to PDK1, and Akt-Ser473 is phosphrylated mainly by mTORC2 [25, 26]. PDK1 is regulated by PTEN through PIP3. Obviously, Akt dysregulation is an important consequence of the loss of PTEN function [27]. Furthermore, the activated Akt plays an important role in cancer formation and progression [28]. In addition to the regulation of apoptosis-associated proteins, it is likely that PTEN/ PI3K/Akt signaling regulates apoptosis by other factors such as NF-κB, p70S6k and glycogensynthase kinase 3 [29, 30].

Researchers found that OMT exerts its anti-tumor effects by regulating cell cycle progression, inducing tumor cell apoptosis, inhibiting angiogenesis, suppressing metastasis and invasion, reversing drug resistance and attenuating chemo radiation-induced toxicity. But the targets of OMT in tumor cells are still unknown. In this study, we observed that OMT could significantly increase the expression of PTEN in MG63 cells. More importantly, we found substantial PTEN in the nucleus after tumor cells were treated with OMT by nucleo-cytoplasmic separation experiment and cell fluorescence staining. By experiments of PTEN RNAi and over-expression of PTEN, we observed that OMT could promote endogenous PTEN expression.

Subsequently studies showed that, OMT made a suppression of PI3K/Akt signaling cascade. The levels of PI3K and phosphorylation of Akt-Thr308 were significantly attenuated by 40 µM OMT. In addition, we also found that, treated by 20 µM OMT for 24 h, MG63 cells were arrested in G1 phase, the level of Cyclin D1and PCNA decreased, and p21 increased. Moreover, OMT could induce MG63and U2OS cell lines apoptosis by up-regulating caspase-3 and Bax expression and down-regulating Bcl-2and inhibit the migration and invasion of them through decreasing MMP2 expression. The antineoplastic activities of OMT on human osteosarcoma cell lines are consistent with the biological function of PTEN in tumor cells. Above data demonstrated that the anti-tumor effects of OMT on human osteosarcoma cell lines is at least partly due to its potential in up-regulating the nucleus PTEN expression and inhibiting PI3K/Akt signaling cascades.

In summary, our data clarified that OMT could inhibit the malignant phenotype of human osteosarcoma cell lines by increasing the expression of PTEN (especially the nuclear PTEN expression) and suppressed PI3K/Akt signaling pathway. Although we got the preliminary results of OMT can inhibit the tumor growth in tumor xenografts mice, Tumor suppression effect need further studies in experimental models *in vivo*. Anyway, the present findings do support that OMT targets PTEN and suppressed the important signaling pathway of PTEN/PI3K/Akt. It provides us anew application in human osteosarcoma, even in PTEN-loss-associated cancer therapies.

## MATERIALS AND METHODS

### Cell culture

MG63, U2OS and HEK293 cells (purchased from Shanghai Maisha Biotech), were cultured in DMEM medium supplemented with 10% fetal bovine serum (Hyclone), under 5% CO_2_ at 37°C.

### MTT assays

Cancer cells (1 × 10^4^/well) were plated in 0.1 ml of the medium containing 10% FBS in 96-well plates. 24 h later, the medium was removed and replaced with 0.1 ml medium containing the indicated concentrations of OMT and incubated for 12, 24, 36, 48 and 60h. At the end of the incubation, the capability of cellular proliferation was measured by the modified tetrazolium salt-3-(4-5 dimethylthiozol-2yl)-2-5diphenyl-tetrazolium bromide (MTT) assay. In this step, 0.01 ml of MTT solution (5 mg/ml in PBS) was added to each well. After a 4 h incubation at 37°C, medium was replaced by 0.15 ml DMSO. After 15 min incubation at 37°C, the optical densities at 490 nm were measured using a Microplate Reader (BIO-RAD).

### Cell-cycle analysis by flow cytometry

MG63 and U2OS cells were incubated with the indicated concentrations of OMT for 24h. After incubation, cells were collected, washed with PBS and then suspended in a staining buffer (10 ug/ml propidium iodide, 0.5% Tween 20, 0.1% RNase in PBS). The cells were analyzed using a FACS Vantage flow cytometer with the Cell Quest acquisition and analysis software program (Becton Dickinson and Co., San Jose, CA). Gating was set to exclude cell debris, doublets and clumps.

### Cell migration and invasion assay

Migration and invasion assays were performed using modified boyden chambers with polycarbonate Nucleopore membrane. Precoated filters (6.5 mm in diameter, 8-μm pore size, Matrigel 100 μg/cm^2^) were rehydrated with 100 μl medium. Then, 1 × 10^5^ cells in 100 μl serum-free DMEM supplemented with 0.1% bovine serum albumin were placed in the upper part of each chamber, whereas the lower compartments were filled with 600 μl DMEM containing 10% serum. After incubating for 18 h at 37°C, non-invaded cells were removed from the upper surface of the filter with a cotton swab, and the invaded cells on the lower surface of the filter were fixed, stained, photographed and counted under high-power magnication.

### Cell apoptosis

By ANNEXIN-V-FITC apoptosis detection kit instructions determination, the specific steps are as follows: cells were washed twice with cold PBS, and the re-suspended with binding buffer cells at a concentration of 1 × 10^6^ cells/ml.Adding 5 μl of ANNEXIN-V-FITC and10 μl of PI. Cells were incubated in dark at room temperature for 15 min. Finally, adding 400 μl binding buffer to each tube and the apoptosis rate was measured by flow cytometrywithin1 h.

### Hochest 33258 staining

Cells were incubated with the indicated concentrations of OMT for 24 h. After incubation, cells were fixed with 4% polyoxymethylene, then washed twice with PBS, incubated with 10 ug/ml hochest 33258 for 5 min at room temperature, then washed with PBS for 3 times. Cells were observed with fluorescence microscope.

### Reverse transcription and quantitative real-time PCR

Total RNA of cells treated with or without OMT for 24 h were extracted using TRIzol (Invitrogen) according to the manufacturer’s protocol. One microgram of total RNA was reverse transcribed to cDNA in a total volume of 20 μl system using a RT reaction kit (Promega). Real-time PCR was performed using an Mx 3000P real-time PCR system (Applied Biosystems) according to the manufacturer’s instruction and SYBR Premix Ex Taq (TaKaRa) as a DNA-specific fiuorescent dye. PCR was carried out for 50 cycles of 95°C for 10 s and 60°C for 30 s. Primer sequences for detection of mRNA expression were synthesized as the Table 1.

**Table 1:** The primers of real-time PCR

Name	Forward primer(5ʹ->3ʹ)	Reverse primer(5ʹ->3ʹ)
Cyclin D1	CCGAGGAGCTGCTGCAAATGGAGCT	TGAAATCGTGCGGGGTCATTGCGGC
P21	AGTCAGTTCCTTGTGGAGCC	AGGAGAACACGGGATGAGGA
PTEN	AGCTGGAAAGGGACGAACTG	ACACACAGGTAACGGCTGAG
PCNA	GCCAGAGCTCTTCCCTTACG	TAGCTGGTTTCGGCTTCAGG
Caspase3	TGTGAGGCGGTTGTAGAAGTT	GCTGCATCGACATCTGTACC
Bcl-2	GGTGAACTGGGGGAGGATTG	GGCAGGCATGTTGACTTCAC
Bax	AGCTGAGCGAGTGTCTCAAG	GTCCAATGTCCAGCCCATGA
MMP2	CGCATCTGGGGCTTTAAACAT	TCAGCACAAACAGGTTGCAG
β-actin	TCGTGCGTGACATTAAGGAG	ATGCCAGGGTACATGGTGGT

All the reactions were repeated at least three times. Gene expression levels were calculated relative to the housekeeping β-actin by using Stratagene Mx 3000P software.

### Western blot analyses

To determine the expression of protein, whole cell extracts (lysate) were prepared from 1 × 10^6^ cells in lysis buffer (20 mM Tris pH7.4, 250 mM sodium chloride, 0.1% Triton-X-100, 2 mM EDTA, 10μg/ml leupeptin, 10 μg/ml aprotinin, 0.5mM phenylmethylsulfonyl fluoride, 4 mM sodium orthovanadate and 1 mM DTT), and 60 μg of the protein was resolved on 10% SDS-polyacrylamide gels. After electrophoresis, the proteins were eletro-transferred to nitrocellulose filters, the membrane (Amersham) was blocked with 5% nonfat dry milk in TBST (20 mM Tris, pH 7.6, 137 mM NaCl, 0.05% Tween-20) for 1 h at room temperature, and the proteins were probed with specific antibodies: CyclinD1, p21, PI3K, PTEN, MMP2, FLAG (Bioworld), PCNA, Akt, phospho-Akt (Thr308), phospho-Akt (Ser 473), Alexa Fluor (488) (Santa Cruz), Caspase3, Bax and Bcl-2 (Neomarker). To assaure equal loading, gels were stripped and reprobed with antibodies againstβ-actin (Kangchen Bio-tech Inc., Shanghai, China). All PVDF membranes were detected by chemiluminescence (ECL, Pierce Technology).

### Transfection of si-RNA

To silence PTEN, MG63cells were transfected with human PTEN siRNA and scrambled control siRNA (Shanghai GeneChem Company) by Lipofectamine 2000 (Invitrogen). According to the manufacturer’s protocol, the final siRNA concentration is 100 nM. The transfection reagent was removed after 12 h and the cells were harvested after 48 h.

### Immunofluorescence analysis

Cells were fixed with 4% paraformaldehyde, permeabilized for 10 min with phosphate-buffered saline containing 0.1% Triton X-100 and blocked with 1% bovine serum albumin. Immunostaining was performed using the appropriate primary and secondary antibodies, and images were acquired using an Olympus fluorescence microscope.

### Tumor xenografts in nude mice

The study protocol was approved by Medical Ethics and Human Clinical Trial Committee. The procedures were approved by institutional animal research ethics committee with reference to the Chinese Community guidelines for the use of experimental animals. Male athymic nude mice (Balb/c nu/nu, 18–20 g) were purchased from Laboratory Animal center of Liaoning Province. The experimental protocol was reviewed and approved by the Ethics Committee of the Shengjing Hospital of China Medical University, Shenyang City, China. All the animals were given free access to sterilized food and water, and were under habituation for 7 d before experimentation. MG63 cells were digested with trypsin and adjust the concentration of suspension. 5 × 10^6^ tumor cells in 100 μl of phosphate-buffered saline were injected subcutaneously into the back of each mouse. The day tumor cells were inoculated was recorded as 0 days. The mice were randomly assigned to saline control group and OMT treatment group, 5 mice each group. The mice were treated with intravenous injection of OMT (100 mg/kg)[31, 32] or saline once a day (days 0–16). The mice were monitored for 16 d after inoculation. The body weights of all mice were recorded throughout the entire experimental period to assess drug toxicity. Any mortality during the course of the study was also recorded. On the 16 day, the mice were sacrificed by cervical dislocation. The weights of the tumors were weighed after dissection.

### Ethical approval

Study received China medical university animal care and use committee approval.
